# The importance of interpreting machine learning models for blood glucose prediction in diabetes: an analysis using SHAP

**DOI:** 10.1038/s41598-023-44155-x

**Published:** 2023-10-06

**Authors:** Francesco Prendin, Jacopo Pavan, Giacomo Cappon, Simone Del Favero, Giovanni Sparacino, Andrea Facchinetti

**Affiliations:** 1 Department of Information Engineering, University of Padova, Padova, Italy; 2 Present Address: Department of Psychiatry and Neurobehavioral Sciences, Center for Diabetes Technology, University of Virginia, Charlottesville, VA USA

**Keywords:** Biomedical engineering, Type 1 diabetes

## Abstract

Machine learning has become a popular tool for learning models of complex dynamics from biomedical data. In Type 1 Diabetes (T1D) management, these models are increasingly been integrated in decision support systems (DSS) to forecast glucose levels and provide preventive therapeutic suggestions, like corrective insulin boluses (CIB), accordingly. Typically, models are chosen based on their prediction accuracy. However, since patient safety is a concern in this application, the algorithm should also be physiologically sound and its outcome should be explainable. This paper aims to discuss the importance of using tools to interpret the output of black-box models in T1D management by presenting a case-of-study on the selection of the best prediction algorithm to integrate in a DSS for CIB suggestion. By retrospectively “replaying” real patient data, we show that two long-short term memory neural networks (LSTM) (named p-LSTM and np-LSTM) with similar prediction accuracy could lead to different therapeutic decisions. An analysis with SHAP—a tool for explaining black-box models’ output—unambiguously shows that only p-LSTM learnt the physiological relationship between inputs and glucose prediction, and should therefore be preferred. This is verified by showing that, when embedded in the DSS, only p-LSTM can improve patients’ glycemic control.

## Introduction

Type 1 diabetes (T1D) is a metabolic disease which impairs insulin production, resulting in an altered glucose homeostasis. As a consequence, subjects must frequently self-administer exogenous insulin, consume corrective/fast-acting carbohydrates (CHO) and follow dietary measures and exercise routines to maintain glycemia into a desired range (i.e., 70–180 mg/dl) along the day^1–3^. Keeping blood glucose (BG) in this tight glycemic range reduces the risk of mortality and the (long and short-term) complications of hyperglycemia (i.e., BG> 180 mg/dL) and hypoglycemia (i.e., BG < 70 mg/dL)^1,4,5^. Minimally invasive continuous glucose monitoring (CGM) sensors have become a commonly used tool by individuals with T1D to keep track of (and eventually correct) their BG levels. These devices provide frequent BG measurements (commonly one every 5 mins) for several days^6–8^. Most of these devices embed visual and acoustic alerts when BG crosses the hypo-/hyperglycemic thresholds, thus helping patients in taking corrective actions to improve their glycemic regulation. However, timely preventive alerts coupled with targeted corrective strategies would be even more helpful to avoid or mitigate the onset of impending, adverse events^9,10^. For this reason, the real-time prediction of future BG levels has a key role in the development of (i) advanced decision support systems (DSS)^11–13^, which are software for helping patients in the decision-making process, and (ii) artificial pancreas systems (APS)^14–16^, which are devices for automating insulin delivery.

As for application of black-box modeling, a key problem of using machine- and deep-learning approaches concerns the interpretability of the outcome. Interpretability represents the degree to which humans can understand the logic beneath a model decision^17^. Whilst machine/deep-learning models can grant accurate performance, their results can be difficult for users to explain and it is often impossible to unveil hidden biases in the datasets or to identify model weaknesses without understanding the decision-making process^18,19^. Moreover, the lack of transparency in their inner logic may hamper the use of these models by arising (legitimate) questions on their trustability and safety.

Modeling BG dynamics makes no exception. Most of the available datasets collected on individuals with T1D present a strong collinearity between insulin administration and carbohydrates intake, i.e., the most commonly employed features for predicting BG levels. As a result, the learning process may sometimes fail and completely misunderstand the effect of these inputs on BG concentration. In recent years, many tools have been developed for providing an interpretation to black-box models and possibly unveil problems in the learning process. Some examples are SHapley Additive exPlanation (SHAP)^20^, Local Interpretable Model-agnostic Explanation (LIME)^21^, Deep Learning Important FeaTures (DeepLIFT)^22^, Model Agnostic Concept Extractor (MACE)^23^ and Generative Adversarial Network (GAN) based methods^24^. These methodologies make algorithms’ predictions individually comprehensible by providing a description of how much each input contributed to the models’ output. Despite of the large number of contributions investigating the use of machine- and deep-learning for BG prediction^25,26^, only a few of them deal with the interpretability of the models^27–29^.

In this work, we aim at discussing some of the issues related to learning glucose prediction models from real data and, in particular, what is the role of intepretability in assessing the safety and usability of these models. Our contribution is twofold: (i) we designed an ad hoc case study to demonstrate the crucial importance that model interpretability has when developing a prediction model for decision-making applications in T1D management; (ii) we propose SHAP (a model-agnostic explainability approach from game theory) as a tool for explaining the output of a glucose prediction model, by showing its efficacy in detecting learning biases.

The case study we designed is a practical one, which consists in choosing a BG prediction algorithm to be embedded into a simple DSS for T1D therapy, with the aim of suggesting corrective insulin boluses to compensate for the suboptimal insulin injection at meal time made by the patient. Being more specific, we trained two machine learning models for BG prediction, which are fed by the same features, are based on a similar structure and provide similar prediction accuracy. In this situation, it is hard to determine which model should be preferred for integration into a DSS.

If we base our choice solely on prediction accuracy, it becomes difficult to establish the superiority of one model over the other, and it may be unclear whether an algorithm can generate BG predictions that lead to safe therapeutic suggestions. However, we will demonstrate that SHAP can be highly beneficial in this process as it reveals that one of these models occasionally generates clinically inaccurate BG predictions, which can result in suboptimal or even unsafe therapeutic suggestions. This happens because the model is unable to accurately estimate how changes in insulin levels affect BG dynamics.

The analysis conducted in this work shows that only the model with accurate feature interpretation is capable of providing appropriate recommendations for corrective insulin boluses.

Interpretability tools like SHAP prove therefore to be key in evaluating prediction models for type 1 diabetes applications. This holds in particular when models are actively used in decision-making or control purposes, where patient safety is a concern. As such, it is desirable that the use of such tools becomes a standard part of model evaluation pipelines.

## Results

The class of models we chose for our case study is long short-term memory (LSTM) neural networks. This model architecture is particularly well fit for time series prediction problems, as it can learn and maintain long and short-term dependencies from data. LSTM networks are not inherently explainable, and thus, they are an appealing case study for the purpose of the present work. We synthesized two different LSTM architectures. In the first one, called non-physiological LSTM (np-LSTM), features are straightforwardly fed into the network. Hence, no specific measure is taken to enforce a physiological interpretation of the inputs. In the second architecture, called physiological LSTM (p-LSTM), a preprocessing layer is interposed between the input and LSTM layer to help the model understanding the correct effect of insulin and CHO on BG level changes. The two models are trained to predict the BG levels ahead in time for two different prediction horizons (PH), i.e., 30 and 60 mins. The training set is composed by the first 6 monitoring weeks of one subject in the OhioT1DM dataset (subject ID 588). Further details on the models are reported in Section “Glucose prediction models”.

The features employed in the models are: the current CGM measurements, *CGM* (mg/dl); the current insulin administration, *insulin* (U/min); the current CHO consumption, *CHO* (g/min). Further details about the dataset and data preprocessing are reported in Section “Dataset and preprocessing”.

Once trained, both models have been assessed according to the following rationale. Step (i): both models are applied on the test set (the last 10 monitoring days of subject ID 588), simulating to receive data in real-time, to evaluate their prediction accuracy which, as anticipated, will be very similar.

Step (ii): predictions obtained in step (i) are interpreted with SHAP to assess whether or not the models are able to correctly explain the output on the basis of available inputs. Step (iii): by exploiting a new tool that allows to re-simulate already collected data, both models have been embedded into a simple DSS designed to suggest corrective insulin boluses and tested in simulated decision-making scenario to evaluate the correctness of insulin suggestions. The following sub-paragraphs describe point-to-point the results obtained for each of these three steps.

### Prediction

The prediction algorithms are tested on a test set consisting of the last 10 days of data of subject ID 588 of the OhioT1DM dataset and their performance are evaluated by using standard metrics.

The first is the mean absolute error (MAE), which is defined as

1 $$\begin{aligned} MAE = \frac{1}{N} \sum _{t=1}^{N} |g(t+PH)-\hat{g}(t+PH|t)|, \end{aligned}$$

with *N* is the number of evaluated samples, *g* is the target output (CGM data in this case) and $\hat{g}(t+PH|t)$ is the PH step ahead BG prediction using the information available up to time *t*. The second metric we use is the root means square error (RMSE):

2 $$\begin{aligned} RMSE = \sqrt{\frac{1}{N} \sum _{t=1}^{N} (g(t+PH)-\hat{g}(t+PH|t))^2}. \end{aligned}$$

Finally, we computed the time gain (TG) which can be considered an average measure of the time anticipation between the target CGM readings and the predicted glucose levels and it is defined as:

3 $$\begin{aligned} TG = PH - delay(g,\hat{g}) \end{aligned}$$

with $delay(g,\hat{g})$ that is quantified as the temporal shift which minimizes the distance between the target and the predicted profiles and it is defined as follows:

4 $$\begin{aligned} delay(g,\hat{g}) = \mathop {\mathrm {arg\,min}}\limits _{j\in {[0,PH]}}\Big [ \frac{1}{N}\sum _{t=1}^{N} (\hat{g}((t+PH|t)+j)-g(t+PH))^2\Big ] \end{aligned}$$

In general, the smaller MAE and RMSE and the larger the TG, the better the algorithms capabilities to forecast glucose levels. Table 1 reports the results obtained with the two LSTM-based models for PH = 30 and 60 min.

Considering the RMSE for a 30-minute prediction horizon, the two algorithms provide similar performance, although a slight improvement in RMSE is granted by the np-LSTM (21.43 mg/dl) with respect to the p-LSTM (21.67 mg/dl). Similarly, for PH = 60 minutes the np-LSTM performs slightly better than p-LSTM (RMSE = 33.16 mg/dl and 33.45 mg/dl, respectively). In both cases, the difference in the performance is less than 1 mg/dl. This was expected, as the only difference between the two architectures consists in the pre-processing layer. Similar considerations can be drawn by looking at the MAE. The np-LSTM provides the best results both for 30-minute and 60-minute PH, even though the performance gap is still small. Finally, Table 1 shows that the average TG is the same for np-LSTM and p-LSTM, i.e., 10 min for PH = 30 and 15 min for 60 mins. This seems to further confirm that the non-learnable pre-processing layer does not influence the overall predicted glucose traces and also it does not add any additional lag to model predictions. Of note, RMSE, MAE and TG are in line with the most of literature’s works, see for instance^30–32^.

**Table 1:** Predictive performance of np-LSTM and p-LSTM for PH = 30 and 60 mins. MAE, RMSE and TG are computed on the test set (last 10 monitoring days) of patient ID 588, OhioT1DM dataset..

Model	MAE (mg/dl)		RMSE (mg/dl)		TG (min)	
	PH = 30 min	PH = 60 min	PH = 30 min	PH = 60 min	PH = 30 min	PH = 60 min
np-LSTM	15.20	23.68	21.43	33.16	10	15
p-LSTM	15.44	23.88	21.67	33.45	10	15

### Interpretation

The general expectation on BG prediction models is that, if a model is able to catch the physiological interpretation of the output, it learns that insulin administration decreases BG levels, whereas CHO consumption has the opposite effect of increasing BG concentration. In practice, two problems usually hinder the learning process: (i) insulin boluses are commonly administered when meals are consumed (the so-called prandial insulin boluses); (ii) the dose of prandial insulin boluses is almost proportional to the quantity of ingested CHO. As a result, black-box algorithms often learn the combined effect of these two inputs, instead of their individual contribution. Many times, the resulting model associates insulin administration to a rise in BG levels^33–35^.

In this work, the interpretation of the four LSTM networks (two structures, two PHs) is provided by resorting to the SHAP summary plot, which details the SHAP value of each individual feature for every sample in the dataset. Each row in the summary plot represents a feature. Each dot in a row represents a specific instance of the training set. The color of the dot indicates whether that data instance is associate with a low (cyan) or high (magenta) value of the feature, with respect to its mean value. The features are ranked by importance on the y-axis (the higher, the more important). The position of an instance on the x-axis indicates its SHAP value, which measures the marginal contribution of a feature on the model’s prediction for a certain instance of the dataset. Hereafter is an example for better clarity. If instances with a high feature value are associated with a large positive SHAP value, then this would suggest that the feature plays a large positive contribution on the model prediction (i.e., the higher the feature, the higher the model output). Lastly, the width of the plots in each row indicates how many instances are associated with that specific SHAP value. Further details on SHAP are reported in Section “Shapley additive explanation”.

**Figure 1:**
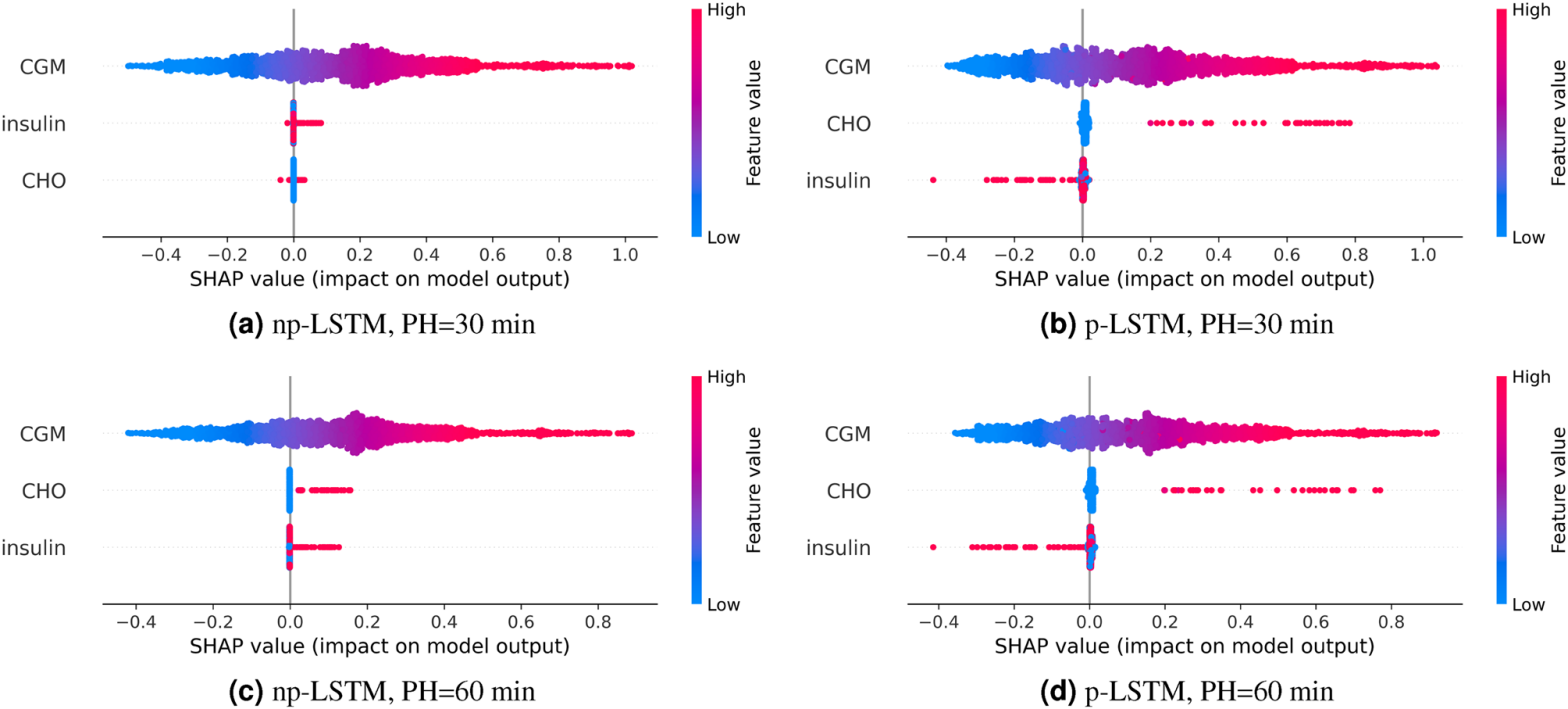
Summary plots of np-LSTM and p-LSTM for different PH.

Figure 1 shows the summary plots corresponding to the both np-LSTM (left side) and p-LSTM (right side) for PHs of 30 (top row) and 60 (bottom row) mins.

Starting from the top-left side (Fig. 1a), i.e., considering the np-LSTM with PH = 30 min, the summary plot reveals that the most important feature is *CGM*. The second most important feature is *insulin*. The impact of *insulin* on the model’s output appears to be weak, as indicated by the relatively small magnitude of the SHAP values associated with this feature compared to those associated with *CGM*. Moreover, the summary plot reveals a counter-intuitive outcome. The SHAP values are not consistently negative for high levels of insulin, meaning that the overall effect of *insulin* is to raise future glucose levels (which is in contrast with the glucose-insulin physiology). Finally, the feature with the least importance is *CHO*, with some values positively affecting BG predictions and others having a negative impact. This is also in contrast with the expected physiological response, as CHO intake is known to increase BG levels in patients with T1D. As for *insulin*, the SHAP values associated with *CHO* ingestion are relatively small compared to *CGM*, implying that the model mainly relies on past CGM readings to predict future BG levels.

This holds true also for the 60-minute np-LSTM (Fig. 1c), where the most important feature is *CGM*. The main difference with respect to the 30-minute np-LSTM, concerns the *CHO* feature which is more important than *insulin* and its contribution to the model output is completely positive (in line with the physiological knowledge). Similarly to the 30-min np-LSTM, the SHAP values associated with insulin are non-negative. This, again, is at odds with physiological interpretation, as high insulin values are expected to lower future BG levels.

In summary, the interpretation of both np-LSTM reveals that BG levels increase with insulin administration, which is the exact opposite of what happens from a physiological point of view.

On the other hand, p-LSTM shows a correct physiological interpretation of the model output for both PH = 30 min (Fig. 1b) and PH = 60 min (Fig. 1d). Also in this case, *CGM* is identified as the most important feature. Both the summary plots indicate that *CHO* is the second most important feature, followed by *insulin*. For both PH, *CHO* has a predominantly increasing effect on predicted BG concentration (as evidenced by positive SHAP values), whereas *insulin* mainly decreases future BG levels (as indicated by negative SHAP values).

### Decision support

For a given PH, p-LSTM and np-LSTM achieve similar prediction results. Therefore, one would expect these models to achieve very similar results when used in a decision-making application. On the other hand, the interpretation provided by SHAP highlights significant differences on how changes in the inputs are connected to changes in the output. In fact, for the np-LSTM, positive values of *insulin* are associated with an increase of *CGM* levels. This behavior is non-physiological and, most likely, it happens because the model learnt the combined effect of insulin and CHO, instead of understanding their individual contribution. The p-LSTM does instead learn the correct signs of these contributions: positive values of *insulin* lead to a decrease in *CGM*, whereas positive values of *CHO* have the opposite effect. Our goal is to verify that, at parity of prediction performance, the model with the correct interpretation should be preferred over the other when used for decision-making and control purposes. To do so, we developed a very simple algorithm to generate corrective insulin boluses (CIB) based on the prediction of future BG concentration. This algorithm is the core of the DSS and it employs the trained LSTM models to assess the effect of different amounts of insulin for the corrective bolus. Then, it selects the insulin dose that leads the predicted future BG level closest to a target level (further details are provided in Section “Corrective insulin bolus algorithm”).

In silico tests are performed by resorting to a simulation framework called ReplayBG (see Section “The simulation tool: ReplayBG”). This tool is used to identify a physiological model that describes a small portion of a real patient’s data. Then, this model is used to simulate (or “replay”) that same portion of data and to perform a retrospective analysis on how our DSS could have changed the glycemic regulation if different therapeutic actions (e.g. the administration of a CIB) were taken. In this study, we chose to model and replay portions of data that are 8 hours long and that begin with an announced meal consumption, so that BG concentration is higher than basal level (and a corrective bolus might be needed). Specifically, we analyzed each postprandial period available in the test set and chose for our retrospective analysis only the portions of data that satisfied the following pre-requisites: (1) no more CHO were consumed during the 8 hours after the first meal intake; (2) no corrective boluses were administered in the original dataset during the 8 hours. These criteria allowed us to find portion of data we considered suitable to fairly assess the effectiveness and safety of the suggestions provided by the DSS. After this selection procedure, we identified 5 postprandial periods suitable for our analysis.

The effectiveness of the suggestions provided by the DSS and the consequent administration of CIBs is then assessed by considering the percentage of CGM samples in different glycemic ranges: within the normoglycemic interval 70-180 mg/dl, called time-in-range (TIR); below 70 mg/dl, called time-below-range (TBR), above 180 mg/dl, called time-above-range (TAR). While the TIR should be maximized, TBR and TAR should be minimized to avoid short- and long-term consequences of T1D, respectively. Besides these standard metrics that are commonly used to evaluate the overall glycaemic control, as reported in the consensus paper^36^, we will consider the amount of suggested boluses and their amount. We would like to minimize these numbers in order to reduce the burden on patients and to avoid possibly risky insulin overload in the organism.

**Figure 2:**
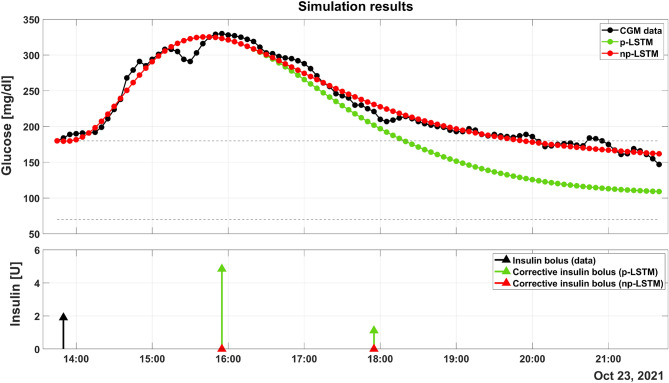
p-LSTM raises two corrective boluses that reduce the time spent in hyperglycemia, while np-LSTM does not suggest any corrective action.

Figure 2 reports the results obtained by applying the DSS based on p-LSTM and np-LSTM in one representative postprandial window. The top panel shows CGM values (black dotted line) outside the normoglycemic range (grey dashed lines) for at least 6 hours (from 14:00 to about 20:00). The green and red dotted lines are the simulated postprandial glucose responses that would have been obtained if the patient had followed the corrective actions suggested by the DSS based on p-LSTM and np-LSTM, respectively. The bottom panel shows the insulin bolus computed by the patient at mealtime (black arrow) and the corrective insulin boluses suggested by p-LSTM and np-LSTM (green and red arrows, respectively). Considering the bottom panel, it can be seen that the DSS based on np-LSTM does not suggest any corrective actions. On the contrary, the DSS based on p-LSTM triggers two CIBs: the amount of the first one is about 5 U and it is suggested around 16:00, the second one is smaller (less than 2 U) and it is around 18:00. The effect of the suggested CIBs on the glucose dynamics are shown on the top panel. Since no boluses are triggered by the np-LSTM, the red dotted line is overlapped to the CGM readings. Differently, the green dotted line shows that the administration of the two corrective actions leads to a decreasing of the time spent in hyperglycemia and a better glycemic control: from 18:00 to 21:00, the green dotted line is completely within the target range.

As shown in Table 2, the DSS based on np-LSTM does not suggest the administration of any CIB neither for 30- nor for 60-min PHs, thus resulting in TBR, TIR and TAR equal to those obtained with original data (0 %, 60.9 % and 39.1 %, respectively). Remarkably, the DSS based on p-LSTM suggests to the patient some corrective actions (1 CIB in median for both 30- and 60-minute PH). All the evaluation metrics, for all the considered PH, benefit from the adoption of the DSS based on p-LSTM. Indeed, considering a 30-min PH, TIR is increased (from 60.9 to 80.7%) and TAR decreases (from 39.1 to 19.3%). Analogously, considering 60-min PH, TIR increases (from 60.9 to 79.2%) and TAR decreases (from 39.2 to 20.8%).

**Table 2:** Results obtained without decision support (No DS) and with two CIB algorithms: one based on np-LSTM (np-LSTM DSS), the other based p-LSTM (p-LSTM DSS).

PH	Therapy	TBR (%)	TIR (%)	TAR (%)	Insulin amount (U)	Number of boluses
30 min	No DS	0 [0–0]	60.9 [42.2–72.4]	39.1 [27.6–57.8]	–	–
	np-LSTM DSS	0 [0–0]	60.9 [42.2–72.4]	39.1 [27.6–57.8]	0 [0–0]	0 [0–0]
	p-LSTM DSS	0 [0–0]	80.7 [63.0–84.9]	19.3 [15.1–36.9]	4.3 [2.1–5.2]	1 [0.5–1.5]
60 min	No DS	0 [0–0]	60.9 [42.2–72.4]	39.1 [27.6–57.8]	–	–
	np-LSTM DSS	0 [0–0]	60.9 [42.2–72.4]	39.1 [27.6–57.8]	0 [0–0]	0 [0–0]
	p-LSTM DSS	0 [0–0]	79.2 [57.8–82.8]	20.8 [17.2–42.2]	2.4 [1.2–2.5]	1 [0.5–1.5]

Results are reported for a PH of 30 and 60 minutes. The results refer to the 5 postprandial periods satisfying the requirements described in Section “Decision support”. Results are reported as median [25th–75th percentiles] computed over these time windows.

## Discussion

In this work we show that not interpreting black-box models could be potentially dangerous in some clinical and practical applications, i.e., when models are actively used to suggest therapeutic actions. We decided to consider as case-of-study the development of a simple LSTM-based DSS that suggests CIBs to mitigate the duration of post-prandial hyperglycemia. Corrective actions are suggested by exploiting the BG predictions provided by the LSTMs. To this end, we designed two ad-hoc LSTM models, np-LSTM and p-LSTM, which rely on the same input features and the same structure. The only difference between the two is a non-learnable, pre-processing layer in p-LSTM, which is placed between the input layer and the hidden LSTM layer. Training machine and deep learning models for accurate glucose prediction typically requires large amount of data. While glucose data can be easily recorded by CGM devices and insulin data by the infusion pump, a common challenge arises with patient’s manually reported information, such as CHO intake. The burden of logging data through a dedicated device or an electronic diary often leads to inconsistency in patients providing their daily diet and treatment information. As an example, subject 567 announced only 31 of the (at least) 141 meals expected during the monitoring period. In fact, when no or few instances of meal intake are provided, the algorithm may be unable to learn any contribution of CHO on future glucose levels. Instead, it may associate the combined effect of insulin and CHO only on the insulin input. Therefore, poor data recording can negatively impact the learning of any model.

To address this issue, we have carefully selected subject ID 588 for conducting our analysis. Among the 12 individuals of the dataset, this patient proved to report the largest number of meal and insulin information during the whole monitoring period (accurately and consistently).

In this context, the key parameter for choosing one among many competing predictive models is prediction accuracy. Therefore, as described in Table 1, we evaluated the models ability to accurately forecast glucose ahead in time in terms of RMSE, MAE and TG. Considering these numbers, it is not completely clear which model to prefer. Both networks provide similar results in terms of accuracy, with the np-LSTM network performing slightly better than p-LSTM.

Nevertheless, when looking at the summary plots, it is clear that the two LSTM models work differently, even though they are fed by the same input features. Glucose levels are expected to rise after CHO intake; vice versa, glucose should decrease after an insulin bolus. The SHAP values in Fig. 1 reveal that p-LSTM has learned this physiological explanation. The other model, np-LSTM, has instead learned an incorrect explanation of insulin and CHO. The SHAP values in Fig. 1 show that *insulin* positively contributes to the model output in np-LSTM for both PH=30 min and PH = 60 min. It means that np-LSTM will forecast a glucose rise after any insulin bolus, even when CHO are not consumed. This happens because of the collinearity between *insulin* and *CHO* in the dataset, which makes it difficult for the learning algorithm to discriminate their individual effect on the output. In conclusion, by looking at the summary plots, the most suitable model for any decision-making application seems to be the p-LSTM.

**Figure 3:**
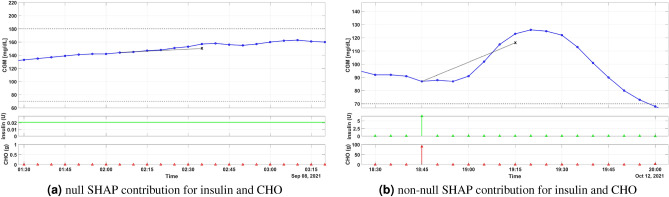
Representative portion of training set at nighttime (**a**) and at meal time (**b**). Top panel: CGM data (blue dotted line), 30-min ahead prediction (black X) provided by p-LSTM. Middle panel: insulin input (continuous line for basal and stem for boluses). Bottom panel: CHO input.

To better understand how p-LSTM relates the input to the prediction, we analyzed some specific instances of the dataset. Firstly, we considered those instances from Fig. 1b for which the SHAP values of insulin and CHO are 0. These instances usually happen during nighttime or during the fasting periods between two consecutive meals, when no CHO are consumed and insulin is infused at basal rate. For these instances, CHO and insulin do not contribute to the model output, and the prediction relies exclusively on CGM. A representative example of these instances is shown in Fig. 3a which reports a portion of the training set during night. CGM data (top panel, blue dotted line) is within the normoglycemic range (black dashed lines) and slowly rising. During nighttime, there are no insulin boluses and the insulin input equals the constant basal rate (middle panel, green line). Also, no meals (bottom panel) have been consumed. The 30-minute ahead in time prediction provided by p-LSTM (top panel, black cross) is close to the target concentration and SHAP attributes a null contribution to insulin and CHO, while a nonnegative contribution is assigned to CGM.

By looking at Fig. 1b, it could also be interesting to focus on the opposite scenario, i.e., by considering all the instances in which both CHO and insulin have a SHAP value significantly different from 0. This condition can happen at meal time, when patients ingest CHO and an insulin bolus is administered. Figure 3b shows a representative example. A meal, most likely a dinner, of about 90 g of carbohydrates was ingested by the patient at 18:45. To avoid glucose exceeding the normoglycemic levels, an insulin bolus of more than 5 U has been administered. Also in this case the 30-minute ahead prediction provided by p-LSTM is close to the target glucose concentration. For this instance, SHAP reveals that: (i) CHO is the most important feature and has a positive contribution to the prediction (SHAP value = 0.76), which is in line with the physiological expectation that CHO ingestion increases glucose levels; (ii) insulin is the second important feature and has a negative contribution (SHAP value = − 0.27); (iii) CGM only marginally contributes to the model prediction (SHAP value = − 0.1).

To enforce previous considerations, thanks to a simulation tool (ReplayBG) we applied both p-LSTM and np-LSTM models to suggest CIBs based on LSTM predictions. Table 2 supports the finding drawn by summary plots, indicating that the most appropriate model for this particular case-study is the one aligned with the physiological meaning, although the p-LSTM model provides slightly lower prediction performance compared to the np-LSTM model.

### Limitations of the SHAP analysis

Despite the interesting results provided by the summary plot, we acknowledge some limitations of SHAP that can lead to erroneous interpretations for particular instances of the dataset. Let’s assume, for instance, that a model is systematically misinterpreting the action of carbohydrates ingestion on future glucose levels because of confounding factors like physical exercise that increases insulin sensitivity and lowers glucose concentration. In this context, the summary plots provided by SHAP would show that a high value of the feature CHO is associated with a low (or even negative) SHAP value. From a physiological viewpoint, this is not in line with the expectations. Similar situations may also happen because of exogenous disturbances affecting the glucose-insulin system, like stress, alcohol intake, illness and menstrual cycle. We acknowledge that SHAP can only grant an explanation about how much each feature relates to the model prediction and it is not to be used for providing causal inference, i.e., for finding the true causes that lead to a specific event. On the other hand, biases in the learning process can only be identified by combining the interpretation provided by SHAP with physiological knowledge.

## Methods

### Dataset and preprocessing

This case-of-study is focused on a single subject (ID 588, female, age 40–60) selected from the OhioT1DM dataset^37^. Briefly, the dataset comprises data from 12 individuals with T1D who were monitored for 8 weeks. Information on glucose and insulin are recorded by CGM devices (Medtronic Enlite CGM) and insulin infusion pumps (Medtronic 530G or 630G), while daily life events are self-reported by users on a smartphone app. Other physiological data, such as skin temperature or heartbeat, are provided by fitness wristbands (Basis Peak fitness for the 2018 cohort, Empatica Embrace for the 2020 cohort). The dataset was collected in a study conducted according to the Declaration of Helsinki, and approved by the Institutional Review Board of Ohio University (protocol number 14-F-12). Informed consent was obtained from all subject involved in the study^38^. The data in the dataset was fully de-identified according to the Safe Harbor method, a standard specified by the Health Insurance Portability and Accountability Act (HIPAA) Privacy Rule^37^.

The subject we selected can be considered as a compliant patient as: (i) it carefully reported a large number of meals and insulin boluses information (e.g., at least 3 meal-related information per day) and (ii) CGM data shows only few missing measurements ( 3% in 10 monitoring weeks), thus allowing a fair assessment of the predictive algorithms and DSSs performance. Also, the patient under investigation is characterized by an elevated TAR (around 46%) and a TIR (around 54%) on the entire test set. The combination of these characteristics make this patient suitable for the aims of this work. Then, patient data are split into a training set, consisting of the first 6 weeks of data, and a test set, which includes the last 10 days of data. The training set is used to train the two LSTM, whilst the test set is used to compute the prediction accuracy of the models. Moreover, we selected a subset of the test set consisting of 8-hours-long post-prandial windows to retrospectively evaluate the insulin corrective actions suggested by the model-based DSS.

Dealing with real data poses some technical issues about synchronization, completeness, and reliability of both stored data and patient’s specific information. The OhioT1DM dataset presents long portion of missing values and the sampling time is not homogeneous. Therefore, all signals were aligned into a uniform time grid with a sampling period of $T_s = 5$ minutes. Any CGM gap in the training set shorter than 30 mins was interpolated with a first order polynomial. No data imputation was performed on the test set.

### Glucose prediction models

Long Short Term Memory neural networks (LSTM) are a suitable choice for time series prediction since they can learn and maintain long and short-term dependencies from data. This kind of network falls in the category of recurrent neural network (RNN), but it overcomes the issue of vanishing-exploding gradient which affects deep RNNs during the training phase. Core elements of an LSTM are the four gates (forget, input, control and output) which compose the so called memory cell. At each time step, these gates decide whether the incoming information is useful or if it must be erased from the cell.

**Figure 4:**
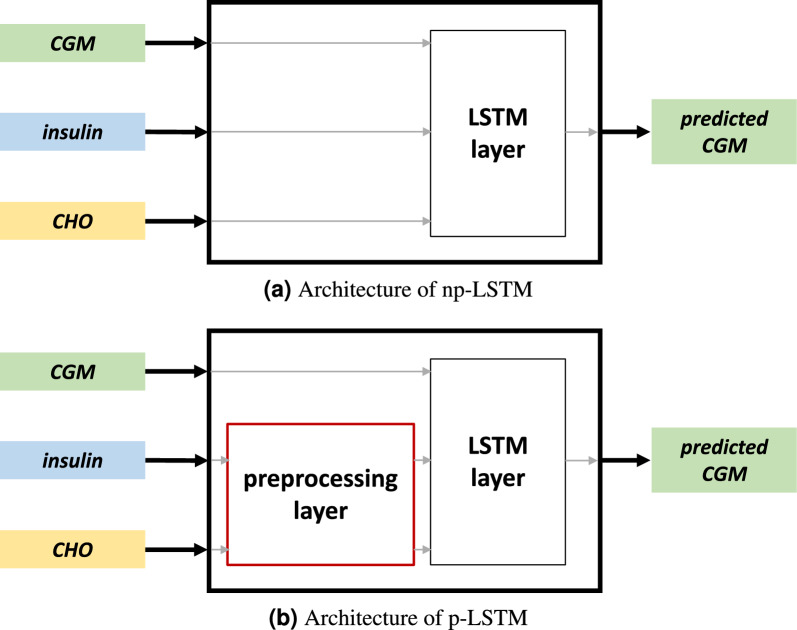
Schematic architecture of np-LSTM (**a**) and p-LSTM (**b**). The only difference between the two structures is the preprocessing layer in (**b**), which is used to enforce a physiological interpretation in the LSTM from insulin and CHO.

For the purposes of our work, we designed two ad-hoc black-box glucose predictive algorithms based on LSTM with the same structure (a single layer of 64 LSTM units): p-LSTM and np-LSTM. As depicted in Fig. 4, these models are fed by the same features and the only difference between the two is represented by the preprocessing layer embedded into p-LSTM. This preprocessing layer, which is placed between the input layer and the LSTM layer, consists of two filters applied to the *insulin* and *CHO* features. These masks resemble the physiological absorption curves characteristics of insulin and CHO, similarly to what described in^39,40^. With these filters, we aim at uncoupling the effects of *insulin* and *CHO* by shifting to their time-action profile on future BG. As showed by the summary plots in Fig. 1, the custom preprocessing layer seems to enable the algorithm in learning the correct effect of insulin and CHO.

#### Preprocessing layer

**Figure 5:**
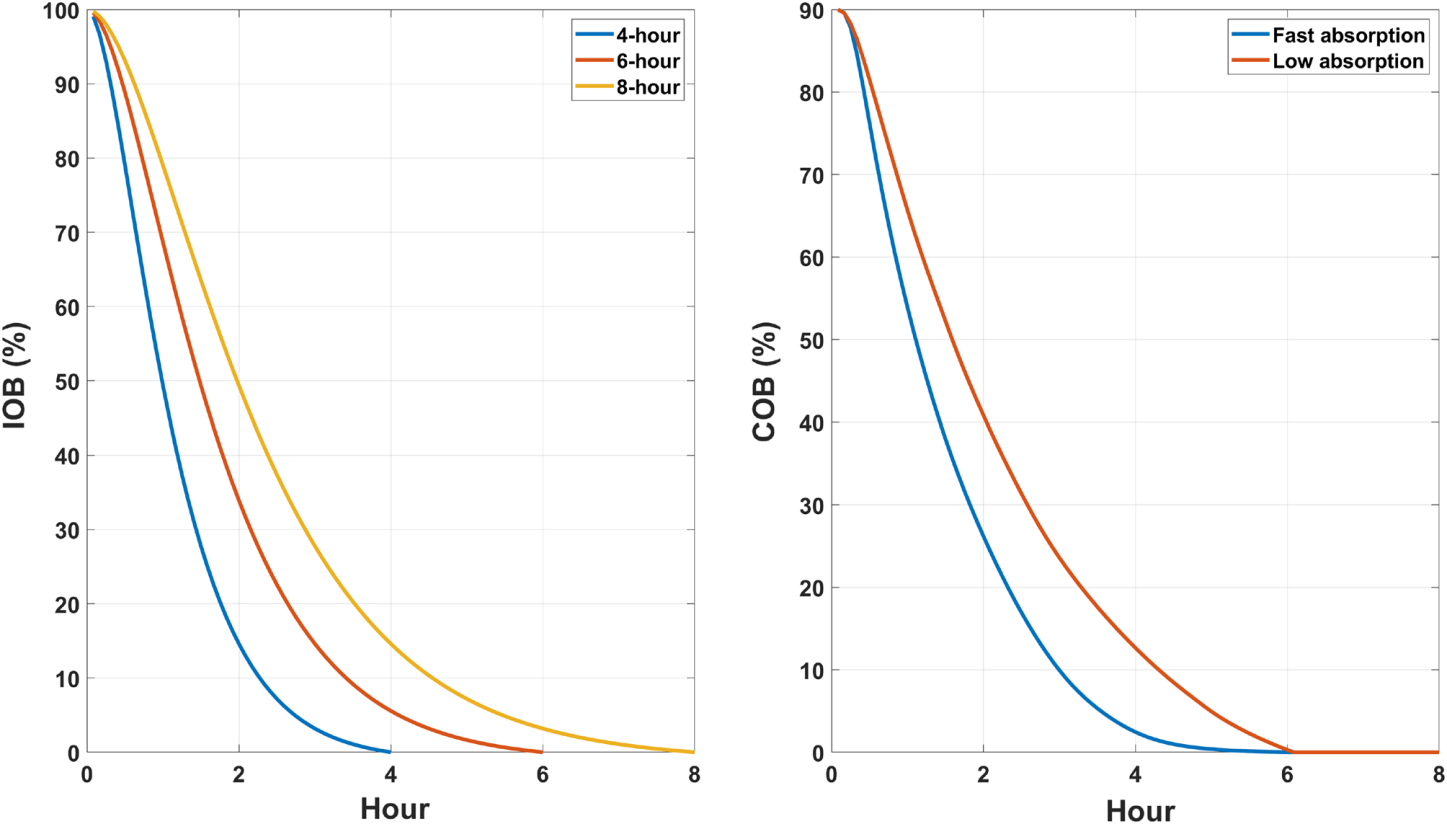
Left panel: insulin decay curves for different durations. Right panel: CHO decay curves for fast and low absorptions. In the preprocessing layer we selected a 6-hour curve for IOB and low absorption curve for COB.

The preprocessing layer represents the main difference among np-LSTM and p-LSTM. This is a custom and non-learnable layer consisting of two filters that provide an approximation of the physiological decay curves of insulin and CHO, as in Fig. 5.

The output of the preprocessing layer is an estimate of both the insulin-on-board (IOB) and carbohydrates-on-board (COB), obtained as the convolution between the input (insulin or CHO) with the filters ($h_{ins}$ and $h_{meal}$), as shown in Eqs. (5) and (6).

5 $$\begin{aligned} IOB(t) = h_{ins}(t)*insulin(t) \end{aligned}$$

6 $$\begin{aligned} COB(t) = h_{meal}(t)*CHO(t) \end{aligned}$$

*IOB*(*t*) can be seen as the amount of active insulin remaining in the body from previous insulin administrations, whereas *COB*(*t*) provides an estimate of the fraction of ingested CHO that has not yet appeared in the circulations. $h_{ins}(t)$ and $h_{meal}(t)$ are the filters, whose parameters are fixed at population values, as in^41–43^. Finally, *insulin*(*t*) and *CHO*(*t*) are the input of the predictive model. From a practical viewpoint, the curves of Fig. 5 represent the impulse responses of the filters ($h_{ins}(t)$ and $h_{meal}(t)$) embedded into the preprocessing layer. The duration of the insulin and meal decay are two additional hyperparameters that have been set to 6-hour for insulin decay and to ‘slow’ for CHO, according to our previous work^27,44^. Of note, similar preprocessing strategies have already been successfully applied in the context of learning black-box models for glucose predictions, see for instance^44–47^, and for control purposes, as in^41,48^.

### Shapley additive explanation

Because of their complexity, LSTM are not inherently interpretable. Nevertheless, several techniques are available for interpreting machine and deep learning models. The interpretation tool we adopted in this work is SHapley Additive exPlanation (SHAP), an approach based on Shapley values that can potentially explain the output of any machine learning model^20^.

The Shapley value method comes from the game theory field. It considers a cooperative game with *M* players and assumes to have the contribution function *v*(*S*) that describes the total expected sum of payoff obtained by a subset of players (*S*). Shapley values fairly distribute the total gain of the game between players. The amount of gain a player *j* receives is given by

7 $$\begin{aligned} \phi (v)_{j} = \phi _{j} = \sum _{S \subseteq {M}\setminus {j}}\frac{|S|!(|M|-|S|-1)!}{|M|!}[v(S\cup {j})-v(S)]. \end{aligned}$$

The difference $v(S\cup j) - v(S)$ indicates the additional contribution that player *j* gave to the subset *S*. Equation (7) defines the Shapley value $\phi _j$ assigned to player *j* as a weighted mean of its additional contributions $v(S\cup j) - v(S)$ to each subset *S* not containing *j* ($S \subseteq {M}\setminus {j}$).

This game theoretical concept can be translated into the context of glucose prediction by understanding the parallelism between players/features and outcome of a game/model prediction.

For a better understanding, let us consider a trivial linear model for predicting blood glucose levels.

8 $$\begin{aligned} \hat{g}(k+PH|k) = f(g(k),i(k)) = \beta _{0}+\beta _{1}g(k)+\beta _{2}i(k) \end{aligned}$$

where *f*(*g*(*k*), *i*(*k*)) is the predictive model that outputs the predicted glucose level—i.e. $\hat{g}(k+PH|k)$- at a certain prediction horizon (PH) ahead in time, once it is fed by CGM at time k, *g*(*k*), and insulin at time k, *i*(*k*). As we are dealing with a linear model, $\beta _{j}$, with $j = 0, 1, 2$, is the weight related to $j-th$ feature (i.e. the model coefficients).

In order to explain the model prediction $f(x^*)$, when the model is fed by a particular instance (denoted by $^*$) of the feature vector $x = x^* = [g(k^*),i(k^*)]$, we have to define a contribution function *v*(*S*), for all possible subset of feature. To quantify this, Lundberg & Lee suggest to use the conditional expectation of the predictive model given a set of feature, i.e $E[f(x)|x_{S}=x^{*}_{S}]$. Following^49^, one can found that the contribution of glucose and insulin at time *k* to the model $f(g(k),i(k)) = \hat{g}(k+PH)$ is given by:

9 $$\begin{aligned} \phi (g(k)) = \beta _{1}g(k)-E[\beta _{1}g] = \beta _{1}g(k)-\beta _{1}E[g] = \beta _{1}(g(k)-E[g]) \end{aligned}$$

10 $$\begin{aligned} \phi (i(k)) = \beta _{2}i(k)-E[\beta _{2}i] = \beta _{2}i(k)-\beta _{2}E[i] = \beta _{2}(i(k)-E[i]) \end{aligned}$$

where *E*[*g*] and *E*[*i*] are the expected value of glucose and insulin. Considering this result, a Shapley value—for a given feature value—can be seen as the deviation of the feature from its mean value, multiply by its weight.

The major advantage of SHAP is that this tool is model-agnostic and it can be applied to deal with any machine and deep learning model. No matter if you are dealing with tree-based models (such as Random Forest or XGBoost) or neural networks (such as feedforward or LSTM models): SHAP unifies several different explanation methods (LIME and Shapley values in the KernelSHAP, DeepLift and Shapley values in DeepSHAP)^20^.

### The simulation tool: ReplayBG

**Figure 6:**
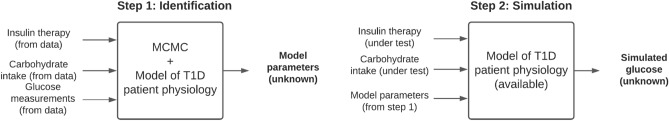
Overview of the two steps of ReplayBG. Step 1: model parameters are identified from CHO, insulin and CGM data already collected by the individual. Step 2: the model parameters identified in Step 1 are given in input to model (6) to simulate the glucose concentration that should have been obtained, in the same individual and in the same time window, by adopting an alternative insulin and/or CHO therapy.

To retrospectively evaluate the goodness of the corrective actions suggested by the LSTM models, we resort to a novel in-silico methodology recently developed by our research team, named ReplayBG^50^. The in-silico framework consisted of two main phases (as shown in Fig. 6): in the first step, a nonlinear physiological model of glucose-insulin dynamics was identified using a bayesian strategy for each selected portion of data. Then, the identified model is used to simulate the post-prandial glucose concentration that would have been obtained by adopting the corrective insulin boluses suggested by the predictive algorithms. The core of this approach is the minimal model described by Bergman et al.^51^, which described the action of plasma insulin on plasma glucose. Such a model has been generalized by adding a model of subcutaneous insulin infusion (describing how exogenous insulin diffuses through plasma^52^ ) and a model of oral glucose assumption (describing how carbohydrates influence BG^53^). Other specific details about such in-silico framework as well as the identification procedure can be found in^50^. Also, an open-source software implementation of the proposed offline Bayesian estimation approach can be found at https://github.com/gcappon/replay-bg.

### Corrective insulin bolus algorithm

As the core of the DSS, the model predictions are exploited to find the optimal corrective insulin bolus to lead BG levels closest to a target glucose concentration. In details, the DSS reasoning works as follows. The algorithm is triggered two hours after a meal intakes and suggests a corrective insulin bolus if BG > 180 mg/dl. The bolus dose is chosen as the $i_n\in \{i_1, \ldots , i_N\}$ that minimizes the following cost function:

11 $$\begin{aligned} J = (\hat{g}_n(k+PH|k)-g_{0})^2 + 10\cdot i_n^2 \end{aligned}$$

where *k* is current time, $\hat{g}_n(k+PH|k)$ is the PH-step ahead prediction provided by the LSTM model when fed by the CGM and CHO values at instant *k* and by the insulin dose $i_n$; $g_{0}$ is a target glucose value (set at 120 mg/dL). The first term of (11) penalizes the distance of the predicted glucose value from its basal value; the second term penalizes the amount of candidate insulin boluses. The minimization problem is solved via grid search in the finite grid of solutions $\{i_1, \ldots , i_N \}\in \mathbb {Q}$.

Quadratic cost functions of this type have been widely employed in artificial pancreas control algorithms to modulate temporary basal insulin rate^54–56^. Of note, the clinical safety and efficacy of the so-formulated minimization problem has been extensively proved for artificial pancreas systems over the last fifteen years^57–59^.

## Data Availability

The data used to support the findings of this study (OhioT1DM Dataset) are available from Ohio University but restrictions apply to the availability of these data, which were used under a Data User Agreement for the current study, and so are not publicly available. Data are however available from Ohio University upon reasonable request at https://ohio.qualtrics.com/jfe/form/SV_02QtWEVm7ARIKIl. The code used for reproducing the results of this manuscript is available at https://github.com/checoisback/interpretable-DL4BGP and https://github.com/gcappon/replay-bg.
